# Transport of the abundant intestinal amino acid glutamine by the enteric pathogen Campylobacter jejuni occurs via GutA (Cj0903), an AGCS family transporter

**DOI:** 10.1099/mic.0.001649

**Published:** 2026-01-12

**Authors:** Ashley Griffin, Jack K. Whitmore, Connor Sharp, Joseph P. Webb, Daniel J. Bennison, Rebecca M. Corrigan, David J. Kelly, Aidan J. Taylor

**Affiliations:** 1 School of Biosciences, University of Sheffield, Sheffield, UK; 2 School of Biological Sciences, University of Reading, Reading, UK; 3 School of Medicine, University College Dublin, Dublin, Ireland

**Keywords:** alanine or glycine:cation symporter (AGCS), ammonium, *Campylobacter*, glutamine, transcriptomics, transporters

## Abstract

Glutamine is the most abundant amino acid in the human body, playing a crucial role in numerous cellular processes. Notably for enteric bacteria, glutamine is abundant in the intestines where it helps to maintain gut health of the host, therefore presenting itself as an accessible nutrient. *Campylobacter jejuni*, a largely non-saccharolytic organism, favours just a few amino acids for growth, and glutamine is particularly efficient as a nitrogen source. Despite this, a glutamine transporter has not been conclusively identified in this important human pathogen. By measuring the global transcriptomic response of *C. jejuni* to replete glutamine conditions, we identified several candidate transporters, ultimately characterising Cj0903, here named glutamine uptake transporter A, as the major glutamine transporter belonging to the alanine or glycine:cation symporter family. We show that this transporter is ubiquitous in thermotolerant *Campylobacter*, demonstrating a conserved ability to utilise exogenous glutamine. In contrast, the ammonium transporter Amt was only present in a subset of *C. jejuni*, and we confirmed that *amt* negative isolates do not effectively utilise ammonium as a nitrogen source.

## Data availability

RNAseq data were deposited with ArrayExpress under accession E-MTAB-11495. Individual samples can be found under the accession numbers SAMEA13207227 - SAMEA13207242.

## Introduction

*Campylobacter* is the leading cause of bacterial gastroenteritis worldwide, with *C. jejuni* responsible for up to 90% of infections [1]. Campylobacteriosis imposes a significant strain on public health and has substantial economic implications: in the UK alone, annual cases are estimated at 300,000 resulting in a financial impact of £700 million [2]. Globally, this expands to estimates as high as 400 million cases per annum [3]. Although infections are typically self-limiting, there is increasing concern about rising antibiotic resistance, highlighting the urgent need for further investigation into this enteric pathogen [4].

*C. jejuni* exhibits unique metabolic features that shape its nutrient utilisation strategies [5]. For example, it lacks a transporter for glucose and does not possess the enzymes glucokinase or phosphofructokinase which are required for glycolysis [6,7]. Additionally, *C. jejuni* lacks the oxidative branch of the pentose phosphate pathway [6,8], further limiting its capacity to derive energy from carbohydrates. Consequently, *C. jejuni* was historically considered non-saccharolytic. However, recent evidence has shown that many isolates have the ability to utilise l-fucose [9,10], and a few can metabolise glucose via the Entner–Doudoroff pathway [11]. Nonetheless, this bacterium relies heavily on alternative sources of nutrients to survive and thrive, predominantly the amino acids serine, aspartate, glutamate, proline, asparagine, and glutamine [12,17], in addition to intermediates from the tricarboxylic acid (TCA) cycle [18,19] and short-chain fatty acids [17,20].

Glutamine is noteworthy as the most abundant free amino acid in the human body [21,22], serving as a major carrier of nitrogen and playing a crucial role in various cellular processes, including nucleic acid and protein biosynthesis [23,24]. Glutamine is an important metabolite for gut health, with one-third of human utilisation occurring in the gastrointestinal tract, providing energy for intestinal cells and maintaining healthy barrier function [25,26]. Humans produce 40–80 g of glutamine per day [27,31], primarily synthesised in the skeletal muscle and subsequently exported via splanchnic circulation to the gut for use by enterocytes and immune cells [32,34]. Intracellular concentrations of glutamine range from 2 to 20 mM [35] with isolated human enterocytes exhibiting a glutamine utilisation rate of 15 µmol min^−1^ g^−1^ dry cell weight [36]. Concentrations in extracellular fluid are around 0.7 mM [37,38], and normal plasma glutamine levels range from 500 to 750 µM, with the small intestine absorbing up to 30% of this glutamine [39,41].

**Fig. 1.**
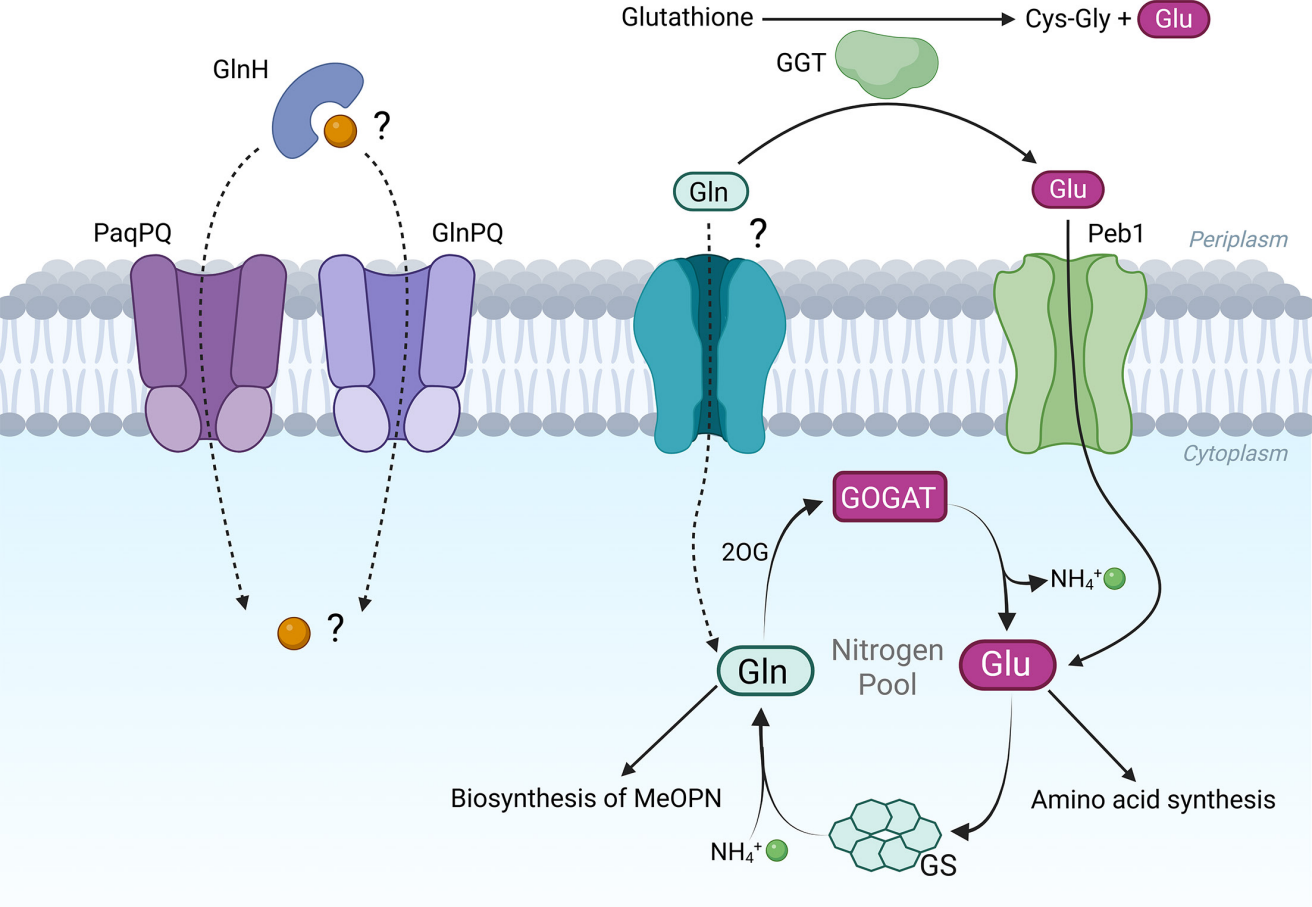
Putative routes for glutamine utilisation in *C. jejuni*. Glutamine (Gln) can be used as a carbon source by conversion to glutamate (Glu) in the periplasm by GGT. GGT also functions to release Glu from host-derived glutathione, producing a Cys-Gly dipeptide which can serve as a sulfur source. Glu is subsequently imported to the cytoplasm via the Peb1 transporter for entry into the glutamine synthetase/glutamate synthase (GS/GOGAT) cycle. Gln may be imported directly, though a transporter is yet to be conclusively identified in *C. jejuni*. ABC type transporter components PaqPQ, GlnPQ, and GlnH have all been implicated in glutamine transport, yet there is a lack of physiological evidence to support this role. Interconversion of Glu and Gln in the GS/GOGAT cycle effectively sequesters and releases cellular nitrogen in the form of ammonium (NH_4_^+^). Beyond their inherent roles as amino acids in proteins, Glu serves as the amino donor for the biosynthesis of all other amino acids, and Gln is utilised in the synthesis of MeOPN, a key component of the CPS.

Given its abundance in the gastrointestinal tract where *C. jejuni* resides, colonising mucosal crypts and adhering to intestinal epithelial cells [42,43], glutamine would appear to be an ideal carbon and nitrogen source. However, while it is generally considered that many *C. jejuni* strains are unable to grow on glutamine as a carbon source, some, including 81-176 [14,44] and 81116 [45], can do so through the activity of γ-glutamyltranspeptidase (GGT), a periplasmic enzyme that hydrolyses glutamine to glutamate, and can also convert host-derived glutathione to glutamate and a cysteine-glycine dipeptide. Experiments by van der Stel *et al*. [46] demonstrated that *ggt* null mutants still exhibit enhanced growth when cultured in media supplemented with glutamine, indicating that glutamine can also be utilised in a GGT-independent manner. Indeed, it is well documented that *C. jejuni* can readily utilise glutamine as a nitrogen source [12,46]. In this case, glutamine is transported into the cytoplasm where it is incorporated into the glutamine synthetase–glutamate synthase (GS-GOGAT) pathway [47]. Glutamine combines with 2-oxoglutarate to form two molecules of glutamate, in effect releasing an atom of nitrogen available for anabolism. Glutamate then donates an amine group for the synthesis of all other amino acids and can sequester excess ammonium in the cell by conversion back into glutamine (Fig. 1). Glutamate can also be a carbon source, through conversion into aspartate and subsequently fumarate, by the action of AspB and AspA, respectively, for entrance into the TCA cycle [13]. Additionally, the amide group of glutamine is utilised directly in the biosynthesis of *O*-methyl phosphoramidate (MeOPN), a key component of the capsular polysaccharide (CPS) [48,49]. Moreover, as glutamine is both readily available in the intestinal niche as an exogenous nitrogen source and acts as the cytoplasmic pool for nitrogen, its direct uptake and utilisation is a highly efficient strategy for *C. jejuni*.

Despite this important role, the mechanism by which *C. jejuni* transports glutamine into the cytoplasm has yet to be fully elucidated. Although there has been some limited evidence that the *C. jejuni* PaqPQ ABC-type transport system accepts glutamine as a solute, there is a lack of physiological data to support its role in uptake [19,50]. Here, we identify and characterise a novel glutamine transporter in *C. jejuni* belonging to the alanine or glycine:cation symporter (AGCS) family (TCDB 2 .A.25). Our results underscore the significant role this transporter plays in the uptake of glutamine in *C. jejuni*, with important implications for its ability to proliferate in the intestinal niche.

## Results

### Utilisation of glutamine by *Campylobacter jejuni*

Utilisation of glutamine as a carbon source first requires conversion to glutamate in the periplasm by GGT, then subsequent import into the cytoplasm by the Peb1 transporter (Fig. 1). Therefore, as has been demonstrated previously, utilisation of glutamine as a carbon source is GGT-dependent. To confirm this, we constructed a *ggt* mutant in *C. jejuni* strain 81-176 and assayed growth on glutamine as the sole carbon source in defined medium (DM) (Fig. S1A, available in the online Supplementary Material). As expected, the *ggt* 81-176 mutant and 11168 wildtype (a naturally *ggt*-negative strain) could not grow on glutamine as carbon source, while growth on glutamate was unaffected. The GGT activity of these and other strains was confirmed by enzymatic assay (Fig. S1B).

We continued to work in strain 11168 where glutamine cannot be used as a carbon source, and therefore use of glutamine as a nitrogen source is dependent on its uptake into the cytoplasm for entry into the GS/GOGAT cycle (Fig. 1). We conducted a series of growth experiments with 11168 wildtype in DM where pyruvate is provided as the carbon source and different amino acids were added as sole nitrogen source. *C. jejuni* is considered to only utilise six amino acids as a nitrogen source: glutamate, glutamine, serine, aspartate, proline, and asparagine. In our DM with pyruvate as carbon source, neither proline nor asparagine supported significant growth (Fig. S1C). Glutamate supported the greatest growth, closely followed by glutamine, then serine and aspartate. Glutamine is therefore a preferential nitrogen source for *C. jejuni*.

### Transcriptomic response of *C. jejuni* to glutamine

To investigate how *C. jejuni* regulates its metabolism in response to glutamine, we conducted a temporal transcriptomic assay in continuous culture. Briefly, 11168 wildtype was grown in glutamine-free DM where a mixture of serine and aspartate was provided as a source of carbon and nitrogen. The culture was grown to steady state, and the first sample was taken at ‘T0’. Immediately thereafter, the culture was spiked with 10 mM glutamine by bolus addition, a concentration representing the midpoint of the physiologically relevant range described above. Further samples were taken at + 5 min (T5), + 20 min (T20) and + 45 min (T45) for comparison to T0. Transcriptomics was performed by RNAseq to identify genes which were differentially expressed in the presence of glutamine.

**Fig. 2.**
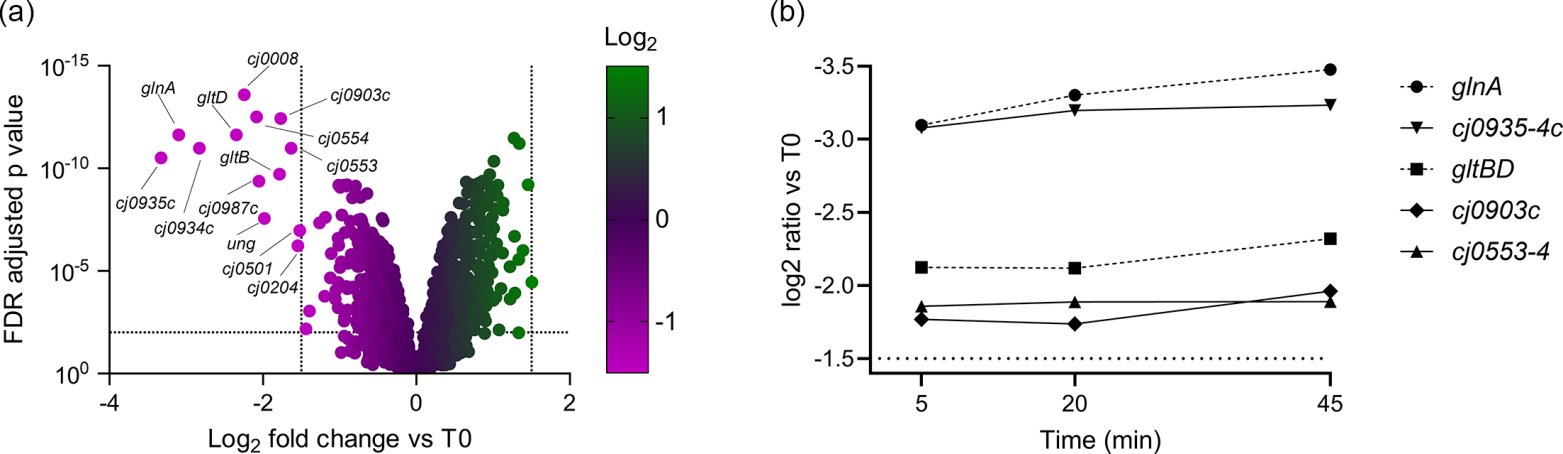
Transcriptional response of *C. jejuni* to glutamine. (**a**) Volcano plot of differentially expressed genes (DEGs) at T5. Dotted grid lines indicate the cut-offs for FDR-adjusted *P* value (<0.01) and log_2_ fold change (>1.5). Significant DEGs are annotated with their gene name or locus tag. (**b**) Temporal transcriptional response of select candidate glutamine transporter (*cj0935-4*; *cj0903c*; *cj0553-4*) and GS-GOGAT cycle genes (*glnA*; *gltBD*).

At T5, 13 genes were significantly downregulated by greater than 1.5 log2 fold change (Fig. 2a). No unique hits were obtained at T20, and only six unique hits were obtained at T45; however, these were genes with no clear relation to glutamine utilisation (Table S3). The temporal pattern of regulation is shown for select genes in Fig. 2(b). Among the genes downregulated at T5 were anticipated genes involved in the GS/GOGAT cycle (*glnA*; *gltBD*) but also several putative inner membrane transporters (*cj0935-4c*; *cj0553-4*; *cj903c*; *cj0204*; *cj0501*; *cj0987c*). *cj0501* and *cj0987c* are pseudogenes and therefore were not investigated further. However, Cj0501 is a homologue of the ammonium transporter Amt, providing a clear link to nitrogen metabolism [51]. Cj0204 (CptA) is a known oligopeptide transporter which is able to import the Cys-Gly dipeptide generated by GGT-dependent conversion of host-derived glutathione to glutamate in the periplasm (Fig. 1) [52]. The remaining three (*cj0935-4c*; *cj0553-4*; *cj903c*) are of unknown function and therefore were candidates for a glutamine transporter.

### Cj0903 is an AGCS family glutamine importer in *C. jejuni*

**Fig. 3.**
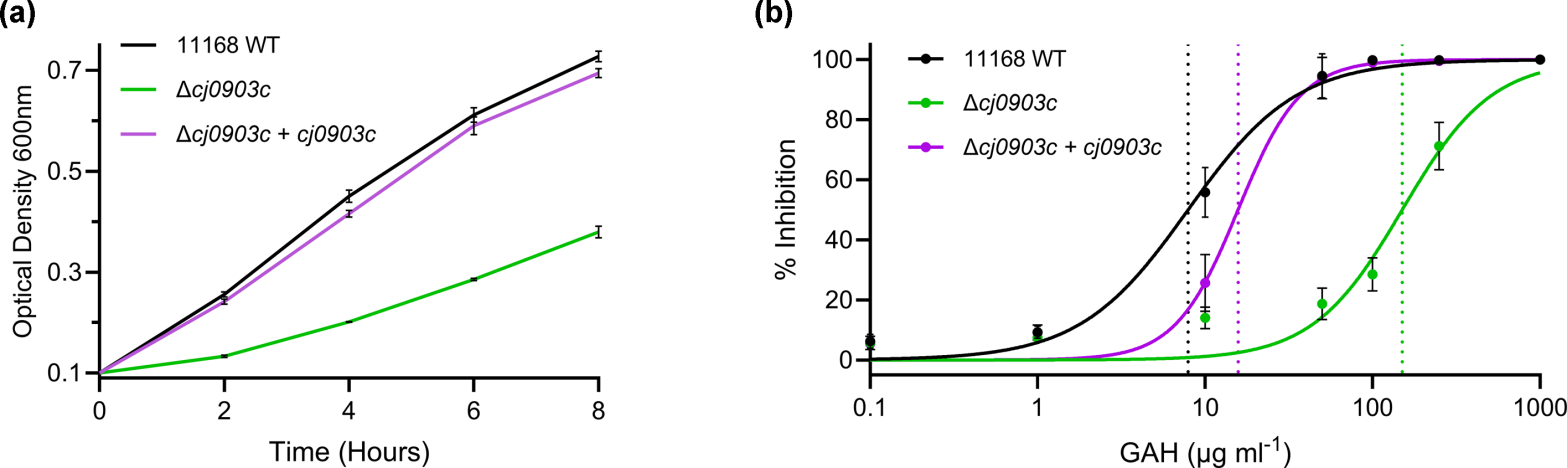
Phenotypic characterisation of Δ*cj0903c* and genetic complement strain: (a) growth curve in DM with glutamine as sole nitrogen source; (**b**) IC_50_ assay with the toxic glutamine analogue l-glutamic acid gamma-hydrazide (GAH). Vertical dotted lines indicate the calculated IC_50_ value (µg ml^−1^ GAH).

We constructed mutants in the three putative transporters identified in our transcriptomic data, as well as in the PaqPQ, GlnPQ, and GlnH ABC type transporter components due to their previously proposed role in glutamine uptake [19,50]. We screened these mutants for growth defects in DM when glutamine was provided as the sole nitrogen source (Fig. S2A, B). The only mutant which displayed a notable phenotype was Δ*cj0903c*, which had a ~50% growth yield reduction on glutamine as nitrogen source compared to wildtype. This phenotype could be restored to wildtype levels by genetic complementation (Figs 3a and S2C). We also constructed double mutants of *cj0903c* with either *paqPQ*, *glnPQ*, or *glnH* but found no change in phenotype, demonstrating that the residual growth in the *cj0903c* mutant was not specifically due to any of these genes (Fig. S2D). To confirm this phenotype was not strain-specific, we also constructed mutants in the homologues of *cj0903c*, *paqP*, *paqQ*, and *glnP* in the *ggt*-positive strain 81-176, as well as double mutants of each with *ggt* (Fig. S1D). The same phenotypic pattern was observed as for 11168 and was found to be GGT-independent.

**Fig. 4.**
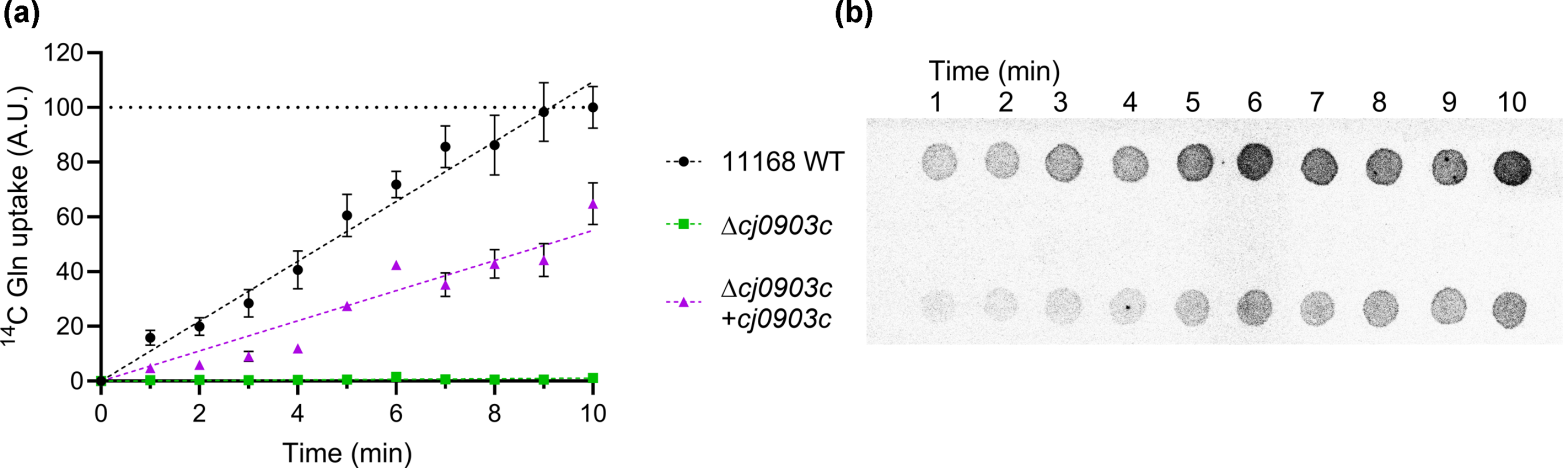
Radiolabelled glutamine uptake assay of Δ*cj0903c* and genetic complement strain. (**a**) Uptake rates of ^14^C glutamine, displayed in A.U., normalised to the maximum signal detected in the wildtype (WT). Values are an average of three replicates. (**b**) Representative phosphorimager image. Rows (top to bottom): *C. jejuni* 11168 WT; Δ*cj0903c*; Δ*cj0903c+cj0903*c.

To support the growth phenotype data, we conducted an IC_50_ assay with the toxic glutamine analogue GAH, a metabolic inhibitor and powerful mutagen, with an IC_50_ value of just 3.92 µg ml^−1^ in *E. coli* [53,54]. GAH requires uptake into the cytoplasm to exert toxicity; therefore, mutants with glutamine uptake defects would be expected to show resistance. The *cj0903c* mutant was significantly more resistant to GAH than wildtype, with a 19-fold increase in IC_50_ value (Fig. 3b). The genetic complement largely restored sensitivity, with an IC_50_ just twofold greater than wildtype. We also assayed a double *paqPQglnPQ* mutant, which likewise had a slight increase in IC_50_ compared to wildtype (Fig. S4A). Lastly, we conducted a radiolabelled uptake assay with ^14^C-l-glutamine which demonstrated a complete absence of transport in Δ*cj0903c*, and restoration to around 50% of the wildtype uptake rate in the genetic complement (Fig. 4). *paqPQ*, *glnPQ*, and *glnH* mutants were also tested by this assay but showed no significant reduction in glutamine uptake compared to wildtype (Figs S3 and S4B). Although we did not perform a full kinetic analysis, given the concentration of radiolabelled glutamine in this assay was in the low micromolar range (1.78 µM; 1 µCi ^14^C), we conclude that Cj0903 is likely the only high-affinity importer for glutamine in *C. jejuni*, with the residual growth of the *cj0903c* mutant in the presence of millimolar glutamine likely due to much lower affinity uptake by other, potentially promiscuous transporters, possibly including PaqPQ given the slight phenotype observed above. We therefore propose to name Cj0903 as GutA (glutamine uptake transporter A).

### Phylogenetic distribution of *gutA* and *amt*

**Fig. 5.**
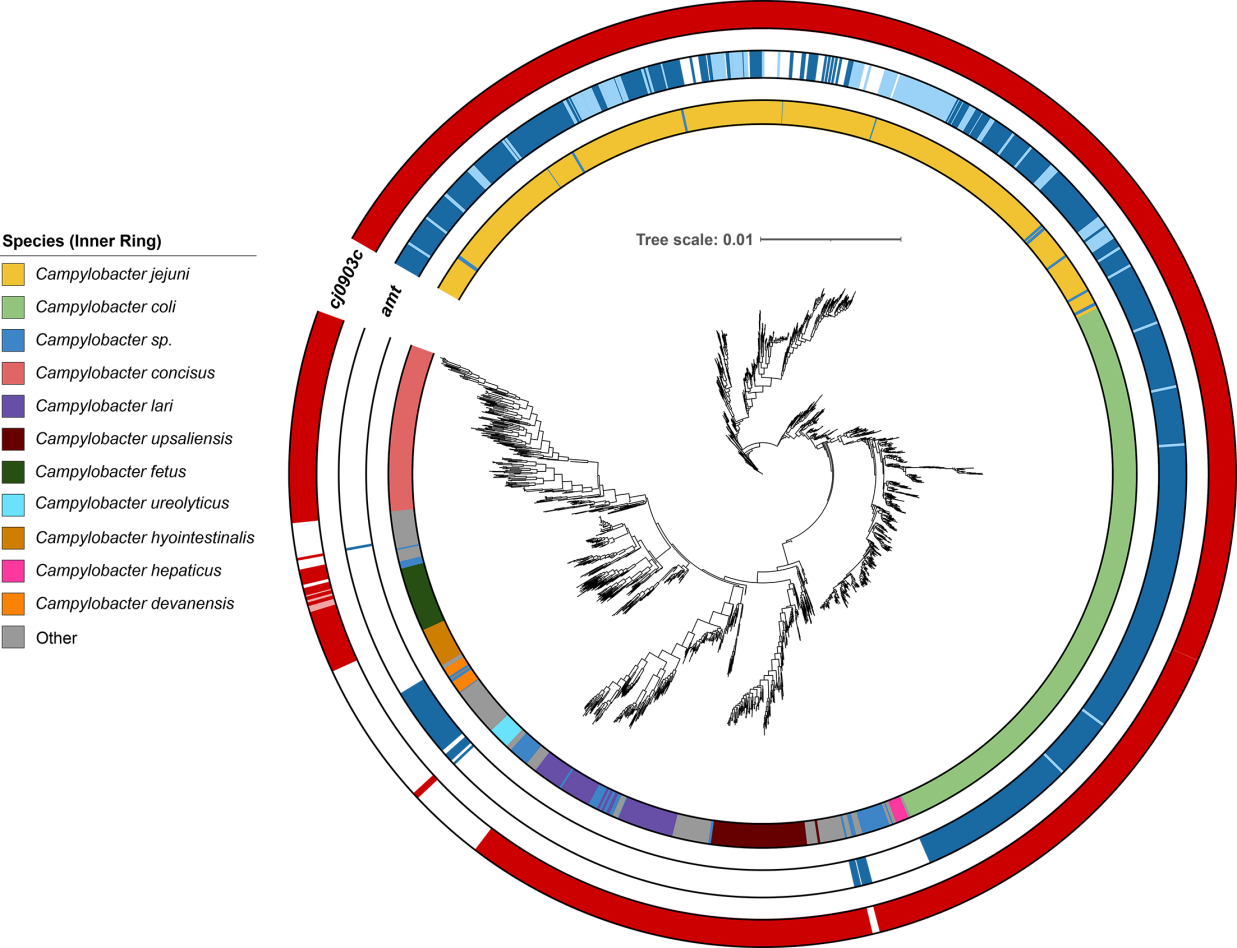
Phylogenetic distribution of *cj0903c* and *amt* in *Campylobacter* spp. The outer and middle rings report the presence (solid colour), absence (white), or pseudogene (light colour) status of *cj0903c* (*gutA*) and *amt*, respectively. The inner ring reports the species of isolate, as indicated in the legend.

After demonstrating the ability of GutA to import glutamine, we set out to investigate if *gutA* is conserved within *C. jejuni*, and across the *Campylobacter* genus. We scanned 6,333 *Campylobacter* genomes for the presence of *gutA* and the putative ammonium transporter *amt* (Fig. 5). In *C. jejuni*, the *gutA* gene is highly conserved, present in >99% of strains (3,234 out of 3,257), with only 9 examples of a pseudogene *gutA*. Of the other *Campylobacter* species analysed, most have a conserved *gutA*, including *C. coli* (97.9%, 1,650 out of 1,685), *C. concisus* (99.6%, 231 out of 232), *C. lari* (100%, 160 out of 160), and *C. upsaliensis* (99.34%, 151 out of 152). Non-functional *gutA* was uncommon in most species, with the exception of *C. fetus* where >25% of strains had a predicted pseudogene. However, we did identify some species of *Campylobacter* which never encode a functional *gutA* gene, though many of these are poorly represented in our dataset, including *C. hyointestinalis* (0 out of 59), *C. davanesis* (0 out of 35), *C. ureolyticus* (0 out of 33), and *C. lanienae* (0 out of 30) (Table S4).

Examining our *Campylobacter* genomes for the presence of *amt* revealed that it is uncommon outside of the thermotolerant *C. jejuni* and *C. coli*. While this putative ammonium transporter is almost ubiquitous and seemingly functional in *C. coli* (99.2%, 1,671 out of 1,685), it is comparatively widely degraded or unidentifiable in *C. jejuni,* with only 52% of strains encoding an intact *amt* gene. Indeed, we were able to demonstrate growth on ammonium as sole nitrogen source by *C. coli* RM1875, but not in the *amt* pseudogene encoding *C. jejuni* NCTC 11168, implying that approximately half of all *C. jejuni* have lost the ability to utilise exogenous ammonium (Fig. S5A).

## Discussion

As a ubiquitous and abundant host-associated amino acid, glutamine represents an ideal nutrient for *C. jejuni*. Although glutamine can be used as a carbon source through conversion to glutamate by GGT prior to uptake, direct uptake of glutamine as a nitrogen source is far more efficient, as glutamine is already the key nitrogen reservoir in the cytoplasm and thus requires no processing (Fig. 1). Indeed, only 31% of *C. jejuni* strains produce GGT, and this enzyme has other known roles in pathogenesis; therefore, we approached our investigation of glutamine utilisation from the perspective of a nitrogen source [55,56].

Through a combination of transcriptomics, mutant phenotyping, and direct uptake measurements, we identified Cj0903 (GutA), a member of the AGCS family, as the likely sole high-affinity glutamine transporter in *C. jejuni*. However, two putative ABC type transporters also had a plausible role in glutamine transport: GlnPQ, annotated as homologous to the prototypical type 1 glutamine transporter, and PaqPQ, as a previous study demonstrated that *paqPQ* mutants have diminished glutamine uptake in *C. jejuni* [50,57]. We therefore constructed mutants in these genes alongside our own candidates but were unable to identify any significant phenotype relating to glutamine transport. A *paqPQglnPQ* double mutant had a small increase in resistance to the toxic glutamine analogue GAH, suggesting that these transporters may be partly responsible for the residual growth of the *gutA* mutant under replete glutamine conditions. Nonetheless, our radiolabelled glutamine uptake assay clearly demonstrates GutA to be the only physiologically relevant uptake system operating at low exogenous concentrations of glutamine. Notably, the *C. jejuni* GlnPQ proteins lack the integral solute-binding proteins (SBPs) of the *E. coli* or *L. lactis* homologues, and neither *glnPQ* nor *paqPQ* is colocalised with a cognate SBP gene in the *C. jejuni* NCTC 11168 genome [57,58]. GlnH is a possible glutamine SBP which may associate with either PaqPQ or GlnPQ, but as yet there is no biochemical evidence for this (Fig. 1). Thus, the true physiological substrate and function of the PaqPQ and GlnPQ transporters in *C. jejuni* remain to be elucidated.

If glutamine is an important nutrient for *C. jejuni in vivo*, one may expect GutA to be important in host colonisation. In fact, over two decades ago, Hendrixson and DiRita demonstrated that a *gutA* transposon insertion mutant in *C. jejuni* 81-176 had a severe colonisation defect in a chick infection model [59]. More recently, *gutA* and *gltBD* were identified among genes downregulated in *C. jejuni* in response to human faecal and chicken caecal extracts, presumably in response to the high glutamine concentrations therein [60]. Taken together with our results, these data suggest that GutA is an important colonisation factor that could provide a target for future inhibition studies. However, although we have shown *gutA* to be almost ubiquitous in thermotolerant *Campylobacter*, high similarity to other AGCS family transporters with potentially disparate substrates meant it was difficult to distinguish GutA homologues in more distantly related bacteria. Only a few members of the large AGCS family have been functionally characterised, notably including a GutA homologue in the distantly related *Staphylococcus aureus* which has also been shown to participate in glutamine transport, despite its annotation as an alanine transporter [61,62]. Therefore, future efforts should focus on sub-typing this family of transporters to enable accurate studies of their phylogenetic distribution, as they may be common amongst diverse bacteria. This is an important consideration for targeting *Campylobacter* GutA therapeutically, as it may be common in beneficial gut bacteria which could suffer collateral damage. In addition, there are diverse mechanisms by which some *Campylobacter* can obtain nitrogen, as discussed below, presenting the risk that targeting glutamine-dependent isolates would simply promote clonal expansion of other genotypes in the host.

Besides *gutA*, our RNAseq data identified the putative transporter operons *cj0935-4c* and *cj0552-4* as glutamine-responsive (Fig. 3). Neither mutant displayed a significant growth phenotype with glutamate or glutamine, indicating a different substrate. *cj0935-4c* encodes an SLC6 type transporter, a sub-family of the neurotransmitter:sodium symporter (NSS) family (TCDB 2 .A.22). The two proteins share a high degree of sequence similarity, suggesting that they may have undergone a duplication event. In eukaryotes, the NSS family transports nitrogen-containing solutes including glycine-betaine, gamma-aminobutyric acid, and various amino acids [63,64]. Characterised examples are sparse in prokaryotes, but an SLC6 homologue in *Bacillus halodurans* can transport several aliphatic and aromatic amino acids [65]. Given the response to glutamine, we suggest it is likely that Cj0935-4 also transports amino acids, though further work is required to characterise these putative transporters. *cj0552-4* encodes three proteins of unknown function: DUF969, DUF979, and DUF2891, respectively. The former two proteins are frequently colocalised and have been suggested to form a transporter for 5-oxoproline, the cyclised degradation product of glutamine and glutamate, due to co-occurrence with the bacterial oxoprolinase [66,67]. Although this provides an obvious rationale for their regulation in response to glutamine, there is no functional characterisation in the literature to support this role. We undertook a preliminary growth experiment with 5-oxoproline as sole nitrogen source and found that *C. jejuni* can indeed utilise it with growth rates similar to glutamine; however, this utilisation was abolished in our *cj0552-4* mutant, strongly suggesting that the DUF969/979 proteins do indeed form a 5-oxoproline transporter (Fig. S5B).

**Fig. 6.**
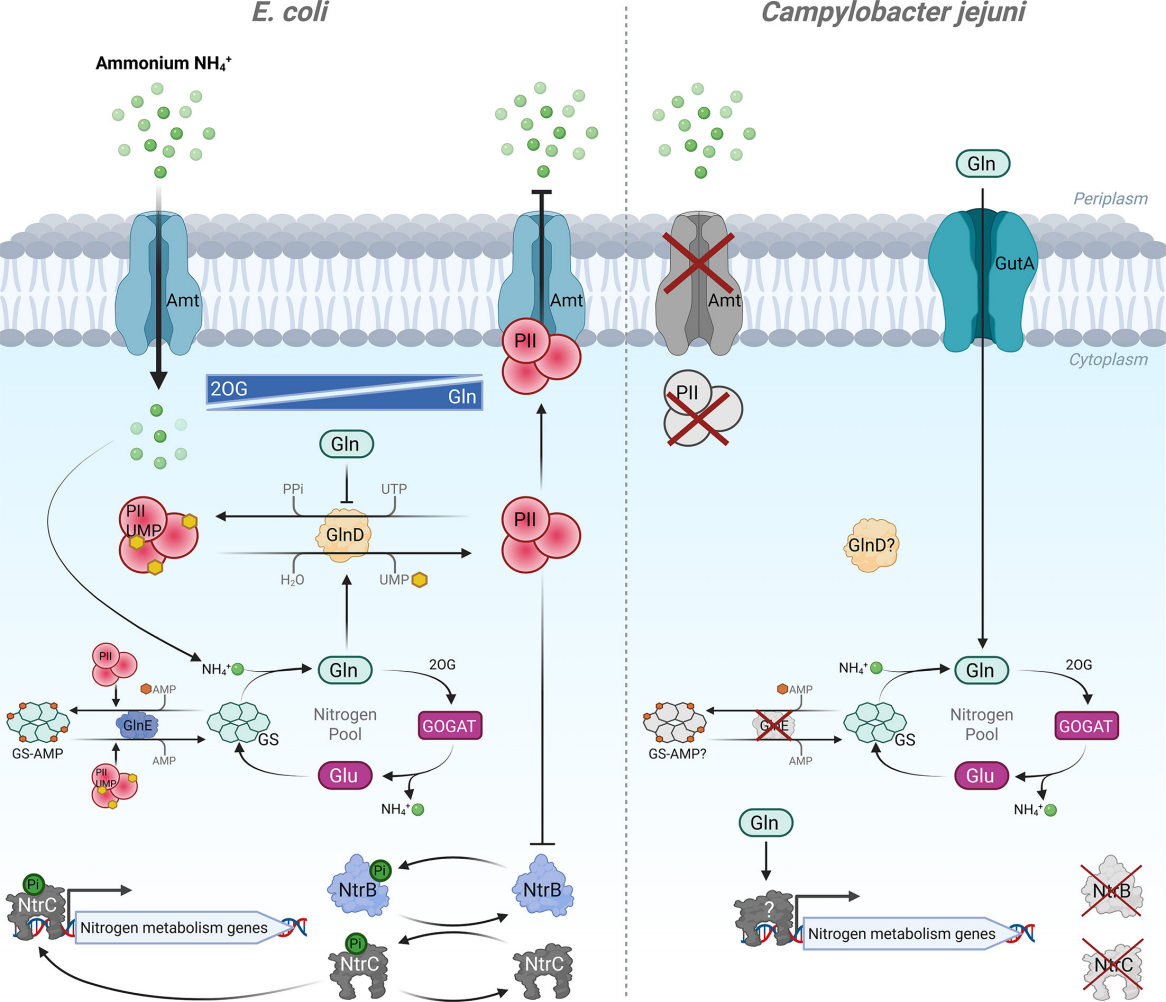
Comparison of *E. coli* nitrogen regulation mechanisms with known elements in *C. jejuni*. *E. coli*: ammonium (NH_4_^+^) is a primary nitrogen source, imported by the Amt transporter when the cellular nitrogen pool is low [sensed as high 2-oxoglutarate (2OG)]. Low Gln levels promote uridylylation of the transducer protein PII (GlnB/K) by uridylyltransferase GlnD. High Gln levels promote inverse activity of GlnD, resulting in unmodified PII which has several modulating functions, including allosteric inhibition of the Amt transporter, reducing glutamine synthase (GS) activity through promoting adenylylation by adenylyltransferase GlnE, and blocking NtrB kinase activity, leading to reduced activation of the transcriptional regulator NtrC and concomitant expression of nitrogen metabolism genes. The overall action of PII and GlnD/E is to maintain homeostasis of cellular Gln levels. Interconversion of Gln and glutamate (Glu) by GS/GOGAT (glutamine synthetase/glutamate synthase) ultimately controls the sequestering and release of NH_4_^+^ for cellular processes requiring nitrogen. *C. jejuni*: canonical factors absent in reference strains of *C. jejuni* include PII, NtrBC, Amt, and GlnE. A protein containing a GlnD-like domain is encoded, but its function is unknown. Therefore, whether post-translational nucleotidylation of GS or other components even occurs in *C. jejuni* is unknown. However, the clear transcriptional response to exogenous Gln reported here does indicate some level of regulation. GutA imports Gln directly and is itself repressed transcriptionally in response to Gln, as are other putative transporters and GS/GOGAT. However, the regulatory transcription factors involved are yet to be identified.

Beyond glutamine, we were also interested in the occurrence of the putative ammonium transporter Amt, as ammonium is a common and often primary nitrogen source for bacteria, yet it is well known that the commonly studied reference strains of *C. jejuni* are unable to utilise it effectively [12]. For example, *C. jejuni* NCTC 11168 encodes a non-functional Amt, and *amt* is entirely absent in *C. jejuni* 81116 and 81-176. Our phylogenetic analysis indicates that the selective pressure to maintain a functional ammonium transporter has been lost in *C. jejuni*, perhaps because direct uptake of glutamine is more energy-efficient and poses less potential toxicity than ammonium. Indeed, the intracellular nitrogen regulation pathways of *E. coli*, which centre around ammonium, are complex and energetically costly, compared to a much simpler model in *amt*-negative *C. jejuni* which appears to lack almost all the regulatory features of *E. coli* (Fig. 6). Nonetheless, our transcriptomic data show a clear multi-gene response to glutamine, mediated by an as-yet unidentified regulatory mechanism. *C. jejuni* encodes few transcriptional regulators, and glutamine-responsive genes identified in our data do not appear to fit within any characterised regulon. However, there are other mechanisms of nitrogen regulation in Gram-positive bacteria and archaea which differ significantly from the *E. coli* paradigm, so it is possible that *C. jejuni* uses an analogous system [68,69].

Comparatively, *C. coli* have maintained a functional copy of *amt*, indicating that utilisation of ammonium provides an advantage in the niche they occupy. Indeed, *C. coli* is commonly associated with pigs, which, owing to their high protein diet and action of the microbiota, can have high millimolar concentrations of ammonium in the gut, an issue garnering much interest due to environmental ammonia release from farms, diarrhoeal disease resulting from dysbiosis of the pig gut, and respiratory conditions caused by high local ammonia gas levels in rearing pens [70]. Some non-thermotolerant *Campylobacter* species, including *C. hyointestinalis*, *C. ureolyticus*, *C. sputorum*, and *C. curvus*, have neither *gutA* nor *amt*, indicating a very different landscape of nitrogen acquisition in these strains. Overall, variation in the nitrogen utilisation strategies of *Campylobacter* species may reflect an ecological adaptation to their preferred host niche.

## Methods

### Bacterial strains and routine culturing conditions

*C. jejuni* (NCTC 11168 or 81-176 DRH212 [71]) cultures were grown in a MACS-VA500 microaerophilic workstation (Don Whitley Scientific Ltd., UK) at 42 °C under microaerobic conditions (10% v/v O_2_, 10% v/v CO_2_, 80% v/v N_2_). *C. jejuni* cells were routinely grown on Columbia agar plates supplemented with 5% v/v defibrinated horse blood, or in liquid cultures of Brucella broth, supplemented with 1% w/v tryptone and 20 mM l-serine (BTS). All *C. jejuni* growth media contained 10 µg ml^−1^ vancomycin, with the selective antibiotics kanamycin, chloramphenicol, and apramycin included, where appropriate, at 50, 20, and 60 µg ml^−1^, respectively. *E. coli* cultures used for cloning were routinely grown aerobically at 37 °C on LB medium, with the selective antibiotics above, and ampicillin at 100 µg ml^−1^, where appropriate.

### DM and continuous culture

DM was used to assay the growth of *C. jejuni* on individual carbon or nitrogen sources. Commercial MEM base (no glutamine, no phenol red: Gibco 51200087) was used with the addition of the following to support growth (final concentrations): 20 mM HEPES; 10 mM sodium formate; 50 µM ferrous sulphate; 100 µM ascorbic acid; 50 µM sodium metabisulphite; 2 mM l-sulfocysteine; 1 mM l-methionine; 2 µM vitamin B12. Where indicated, sodium pyruvate was provided as carbon source at 20 mM. Individual amino acids were added as nitrogen source at 5 mM. Unless otherwise stated, all single point growth measurements were taken at 8 h after inoculation. For continuous culture experiments, the same media formulation was used, in a final volume of 885 ml, utilising a Labfors 3 bioreactor (Infors) with the following settings: dilution rate, 0.2 h^−1^; stirring, 350 r.p.m.; gas flow, 0.5 l min^−1^; gas mix, 5% v/v O_2_, 10% v/v CO_2_, 85% v/v N_2_; temperature, 37 °C. The culture was considered to have achieved steady state after five volumes of turnover.

### Genetic manipulation of *C. jejuni*

Knock-out deletion mutants were generated by replacing the majority of the ORF with an antibiotic resistance cassette (kanamycin, chloramphenicol, or apramycin, amplified from pJMK30, pAV35, or pRRA, respectively), utilising spontaneous double-crossover recombination to delete the majority of the ORF. Mutation and complementation plasmids were generated by an isothermal assembly method, as recently reported in detail [72]. In brief, upstream and downstream flanking regions of knock-out deletion target genes were amplified by PCR from genomic DNA using primers with adapters homologous to either the antibiotic resistance cassette or plasmid vector (pGEM3ZF), as appropriate. Four DNA fragments (upstream flank, downstream flank, antibiotic resistance cassette, and HincII linearised pGEM3ZF) were combined in an isothermal assembly reaction using HiFi DNA Assembly Master Mix (NEB). The generated mutagenesis plasmids were transformed into *E. coli* DH5α and successful recombinants screened by PCR. *C. jejuni* was made competent by washing in a solution of 9% w/v sucrose and 15% v/v glycerol and transformed with purified mutagenesis plasmids by electroporation (1.5 kV, 5 ms). Successful transformants were isolated on appropriate selective media and verified by PCR screening to confirm correct recombination onto the genome. Genetic complementation followed a similar process, utilising the *Campylobacter*-specific complementation vector pC46 to reintroduce the gene of interest into the genome at the *cj0046* pseudogene locus. The gene of interest was amplified by PCR from genomic DNA with primers containing BsmBI restriction sites, enabling ligation into linearised pC46 to generate a complementation plasmid. Strains and primers used in this study are listed in Tables S1 and S2.

### GGT activity assay

GGT activity of isolates was measured by the production of 3-carboxy-4-nitroaniline, monitored as an increase in absorbance at 405 nm. Briefly, overnight cultures were pelleted, washed twice in 50 mM Tris-HCl pH 7.6 and, finally, resuspended in 1× amine-free BugBuster Protein Extraction Reagent (Novagen) to create a cell-free extract, following the manufacturer’s instructions. An assay mix was prepared of 2.9 mM l-glutamic acid γ-(p-nitroanilide) and 100 mM glycylglycine in 50 mM Tris-HCl pH 8.2. In a 96-well plate, 50 µl of cell-free extract was combined with 150 µl of assay mix and incubated at 37 °C, with the increase in absorbance at 405 nm measured over 20 min. The total protein content of cell-free extracts was determined by Lowry assay, and a specific GGT activity was calculated in µmol min^−1^ mg^−1^ total protein, using the extinction coefficient of 3-carboxy-4-nitroaniline at 405 nm of 10.4 mmol^−1^ cm^−1^.

### Transcriptomics

Immediately following extraction from the bioreactor, culture samples were spun at 15 *g* for 2 min at 4 °C, the supernatant removed and cell pellets flash-frozen in liquid nitrogen prior to storage at −80 °C. Frozen cell pellets were sent to Genewiz (Azenta) for standard RNAseq analysis, involving RNA isolation, rRNA depletion, transcript library preparation, and paired-end sequencing on an Illumina Hi-Seq platform. Differential gene expression was analysed using the Galaxy web-based platform [73]. Bowtie 2 was used to align trimmed reads to the *C. jejuni* NCTC 11168 reference genome [74]. Numbers of mapped reads aligned to each gene were counted using HTSeq [75]. Raw counts were converted to log2 counts per million using the LIMMA voom transformation, and significantly DEGs were analysed using the LIMMA package in R [76]. RNAseq data were deposited with ArrayExpress under accession E-MTAB-11495.

### GAH IC_50_ assay

To determine the IC_50_ of GAH, a 9-point 10-fold dilution series of GAH was prepared in DM with glutamate as nitrogen source, with the highest concentration as 1,000 µg ml^−1^. Cultures were incubated for 16 h, and the end-point OD_600nm_ values were measured and converted to a % inhibition value relative to controls. IC_50_ values were calculated by fitting the data to a four-parameter nonlinear regression model ‘inhibitor vs response’ in GraphPad Prism.

### Radiolabelled glutamine uptake assay

Mid-log DM cultures (20 mM sodium pyruvate, 5 mM l-glutamate) were harvested and washed twice with in DM media (without carbon or nitrogen source) and normalised to an OD_600nm_ of 1.0 and stored on ice. 100 µl of cells were transferred into 900 µl DM (20 mM sodium pyruvate) and incubated at 42 °C for 3 min. A 1 µCi solution of ^14^C labelled l-glutamine (1.78 µM) was prepared, and 10 µl was added to the 1 ml culture, together with 50 µl of 1 mM l-glutamine, and incubated at 42 °C. Every minute for 10 min, 100 µl samples were taken and quenched with 10 mM final l-glutamine (stop buffer). Samples were centrifuged and washed 3 times in 1 ml stop buffer before finally being resuspended in 10 µl of stop buffer and spotted onto Whatman filter paper. Spots were imaged after 48 h using a phosphorimager. The immediate local background of each spot was normalised using an adapted DRaCALA equation [77].

### Bioinformatics

To analyse the distribution of *gutA* and *amt*, 6,333 *Campylobacter* genomes were accessed from RefSeq, including only genomes which have been annotated by RefSeq and excluding atypical genomes, metagenome assembled genomes, and genomes from large multi-isolate projects. Genes were identified using tblastn with the query sequences for *gutA* (*cj0903c*) and a reconstructed *amt* (*cj0501*) from *C. jejuni* NCTC 11168, using a >50% identity and >70% alignment cut-off [78]. Pseudogene assignments were derived from the RefSeq annotation of each genome. To calculate a phylogeny, genus-wide core genes were used. As the dataset was dominated by *C. jejuni* and *C. coli*, these species were subsampled to remove bias. Mashtree was used to calculate a phylogeny individually for *C. jejuni* and *C. coli* [79]. These trees were then subsampled to 1,000 samples for each species using treemmer [80]. Genus-wide core genes were identified using Panaroo which identified 39 core genes (present in ≥95% of strains) [81]. These genes were aligned using Clustal Omega, and a phylogeny was calculated using IQ-TREE2 (using the model GTR+F+I+R4) and ClonalFrameML [82,84]. The final phylogeny was visualised using iTOL [85].

## Supplementary material

10.1099/mic.0.001649 Uncited Fig. S1.

## Abbreviations

AGCS: alanine or glycine:cation symporter
CPS: capsular polysaccharide
DEGs: differentially expressed genes
DM: defined medium
GAH: l-glutamic acid gamma-hydrazide
GGT: γ-glutamyltranspeptidase
GutA: glutamine uptake transporter A
MeOPN: *O*-methyl phosphoramidate
NSS: neurotransmitter:sodium symporter
SBPs: solute-binding proteins
TCA: tricarboxylic acid
