# Supplementary material for: Transport of the abundant intestinal amino acid glutamine by the enteric pathogen Campylobacter jejuni occurs via GutA (Cj0903), an AGCS family transporter
Source: Microbiology (Reading). 2026 Jan 12;172(1):001649. doi: 10.1099/mic.0.001649 (PMC12795558; doi:10.1099/mic.0.001649)
Supplement: Uncited Fig. S1. [file mic-172-01649-s001.pdf]

Transport of the abundant intestinal amino acid glutamine by the enteric pathogen *Campylobacter jejuni* occurs via GutA (Cj0903), an AGCS family transporter

Ashley Griffin [1#], Jack K. Whitmore [2#], Connor Sharp [2], Joseph P. Webb [1], Daniel J. Bennison [1], Rebecca M. Corrigan [3], David J. Kelly [1*] and Aidan J. Taylor [2*]

1: School of Biosciences, University of Sheffield, Sheffield, UK
2: School of Biological Sciences, University of Reading, Reading, UK
3: School of Medicine, University College Dublin, Dublin, Ireland

Supplementary material

Contents:

Supplementary Figure 1: Growth of *C. jejuni* on different amino acids, and GGT activity.

Supplementary Figure 2: Growth of *C. jejuni* 11168 on different amino acids.

Supplementary Figure 3: Full phosphor-images of radiolabelled glutamine uptake.

Supplementary Figure 4: GAH IC_{50} data and ^{14}C glutamine uptake rates.

Supplementary Figure 5: Growth of *Campylobacter* on ammonium and 5-oxoproline.

Supplementary Table 1: Strains used in this study.

Supplementary Table 2: Primers used in this study.

Supplementary Table 3: RNAseq significant DEGs.

Supplementary Table 4: Distribution of *gutA* and *amt* in *Campylobacter* spp.

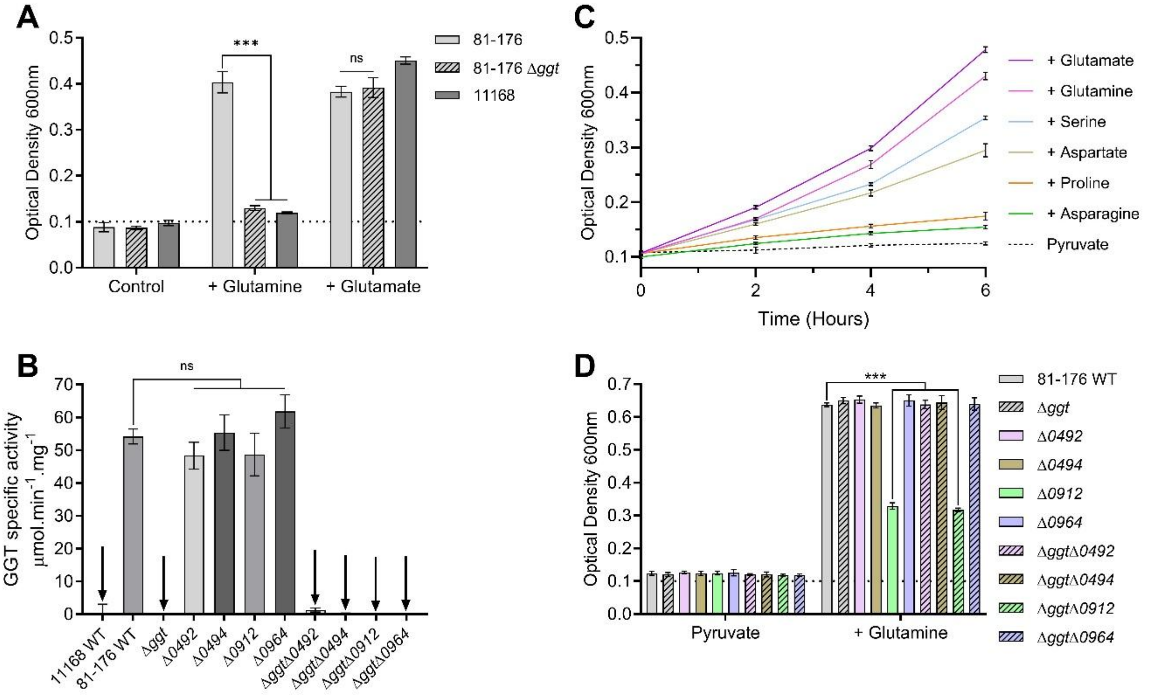

Supplementary Figure 1. A) Growth of *C. jejuni* 11168, 81-176, and 81-176 Δ*ggt* on either glutamine or glutamate as sole carbon source. **B)** Growth of *C. jejuni* 11168 on individual amino acids as sole nitrogen source, with pyruvate as carbon source. **C)** GGT activity of *C. jejuni* 81-176 strains, determined by enzymatic assay. *C. jejuni* 11168 acts as a GGT-negative control. Arrows indicate negligible activity detected. **D)** Growth of *C. jejuni* 81-176 strains on glutamine as sole nitrogen source, with pyruvate as carbon source.

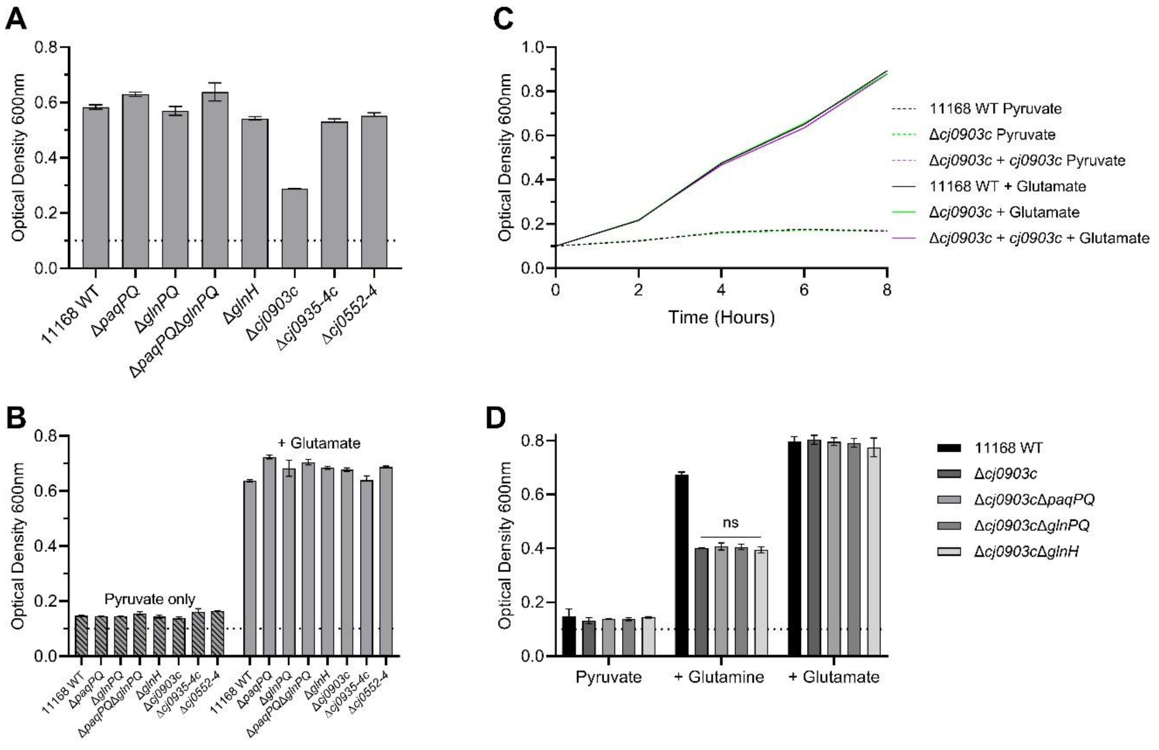

Supplementary Figure 2. A) Growth of *C. jejuni* 11168 strains on glutamine as sole nitrogen source, with pyruvate as carbon source, or **B)** controls. **C)** Control growth curves of *C. jejuni* 11168 *cj0903c* strains on pyruvate-only or glutamate. **D)** Growth of *C. jejuni* 11168 double mutants in Δ*cj0903c*.

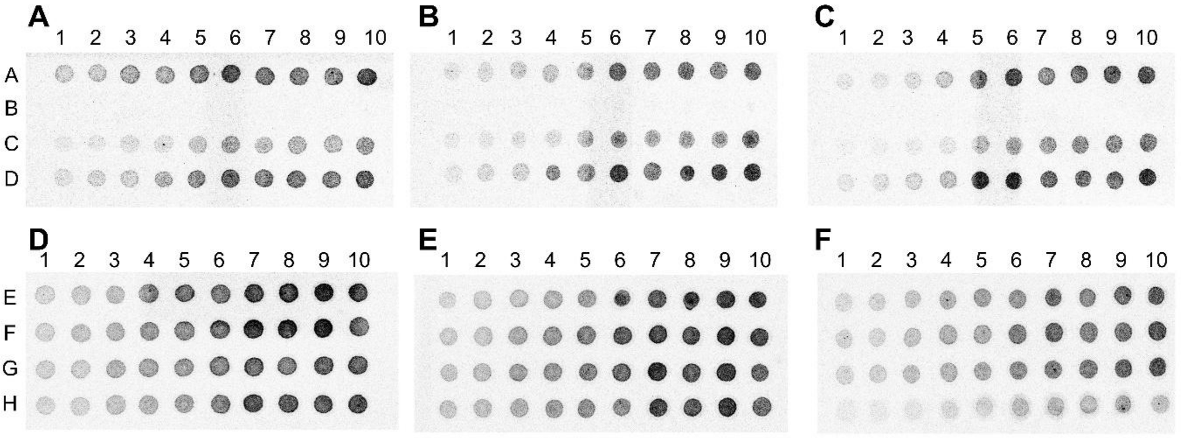

Supplementary Figure 3. Full phosphor-images of radiolabelled glutamine uptake. Columns (left to right): Time, 1 - 10 min (1 min intervals). **A-C)** 3 replicates. Rows A;B;C;D: *C. jejuni* 11168 WT; Δ*cj0903c*; Δ*cj0903c* + *cj0903c*; Δ*glnQ*. **D-F)** 3 replicates. Rows E;F;G;H: *C. jejuni* 11168 WT; Δ*paqPQ*; Δ*glnH*; Δ*glnP*.

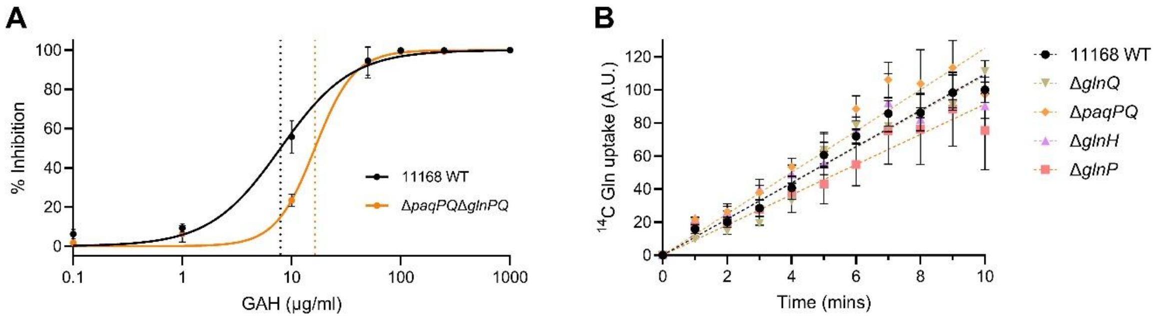

Supplementary Figure 4. A) IC_{50} assay of L-Glutamic acid gamma-hydrazide (GAH) with *C. jejuni* 11168 WT and Δ*paqPQ*Δ*glnPQ*. Vertical dotted lines indicate the calculated IC_{50} value (µg/ml GAH). **B)** Uptake rates of ^{14}C glutamine for other strains tested with no significant change from WT.

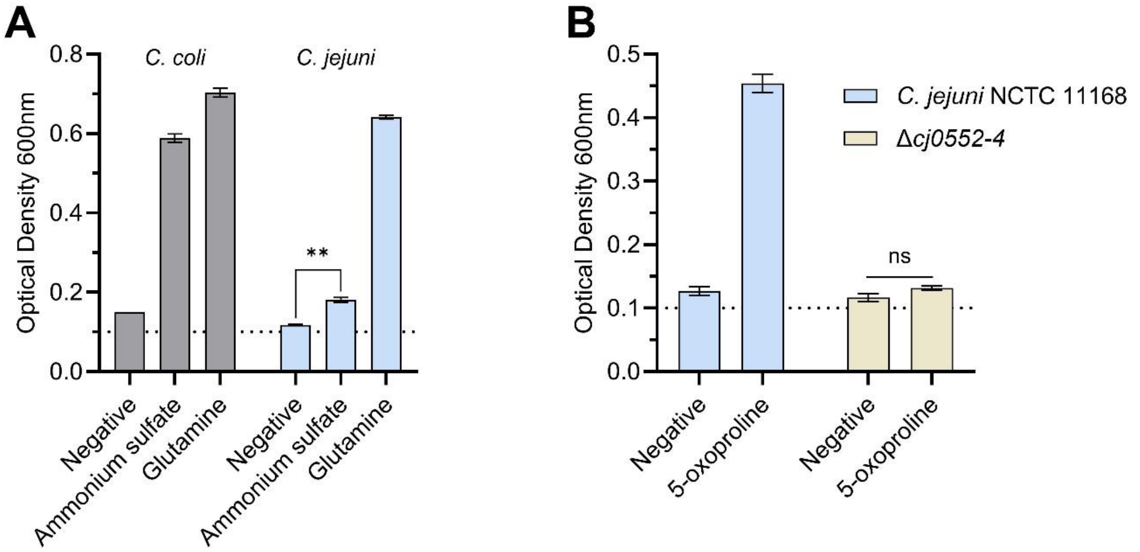

Supplementary Figure 5. Growth in DM with pyruvate as carbon source of **A)** *C. coli* RM1875 and *C. jejuni* NCTC 11168 on ammonium sulfate as sole nitrogen source, and **B)** *C. jejuni* NCTC 11168 wildtype and Δ*cj0552-4* on 5-oxoproline as sole nitrogen source. Dotted lines indicate the starting inoculum.

Supplementary Table 1: Strains used in this study.

Strain name	Mutations	Source
C. jejuni NCTC 11168 WT	none	Skirrow (1977)
Δcj0903c	Δcj0903c (Kan)	This study
Δcj0903c + cj0903c	Δcj0903c (Kan) : pC46 (Cat)	This study
Δcj0935-4c	Δcj0935-4c (Kan)	This study
Δcj0552-4	Δcj0552-4 (Kan)	This study
ΔpaqPQ	Δcj0467-9 (Kan)	This study
ΔglnPQ	Δcj0901-2 (Kan) : Δcj0940 (Apr)	This study
ΔglnH	Δcj0817 (Kan)	This study
ΔpaqPQΔglnPQ	Δcj0467-9 (Kan) : Δcj0901-2 (Cat) : Δcj0940 (Apr)	This study
Δcj0903cΔpaqPQ	Δcj0903c (Kan) : Δcj0467-9 (Cat)	This study
Δcj0903cΔglnPQ	Δcj0901-3c (Kan) : Δcj0940 (Apr)	This study
Δcj0903cΔglnH	Δcj0903c (Kan) : Δcj0817 (Cat)	This study
C. jejuni 81-176 WT	DRH212; streptomycin resistant	Hendrixson, Akerley and DiRita (2001)
Δggt	ΔCJJ81176_0067 (Cat)	This study
Δ0492 (ΔpaqP)	ΔCJJ81176_0492 (Kan)	This study
Δ0494 (ΔpaqQ)	ΔCJJ81176_0494 (Kan)	This study
Δ0912 (Δcj0903c)	ΔCJJ81176_0912 (Kan)	This study
Δ0964 (ΔglnP)	ΔCJJ81176_0964 (Kan)	This study
ΔggtΔ0492	ΔCJJ81176_0067 (Cat) : ΔCJJ81176_0492 (Kan)	This study
ΔggtΔ0494	ΔCJJ81176_0067 (Cat) : ΔCJJ81176_0494 (Kan)	This study
ΔggtΔ0912	ΔCJJ81176_0067 (Cat) : ΔCJJ81176_0912 (Kan)	This study
ΔggtΔ0964	ΔCJJ81176_0067 (Cat) : ΔCJJ81176_0964 (Kan)	This study

Abbreviations
Kan; kanamycin selection
Cat; chloramphenicol selection
Apr; apramycin selection
Δ; knockout mutation
pC46; genetic complementation vector

Skirrow MB. Campylobacter enteritis: a "new" disease. Br Med J. 1977 Jul 2;2(6078):9-11. doi: 10.1136/bmj.2.6078.9. PMID: 871765; PMCID: PMC1631297.

Hendrixson DR, Akerley BJ, DiRita VJ. Transposon mutagenesis of Campylobacter jejuni identifies a bipartite energy taxis system required for motility. Mol Microbiol. 2001 Apr;40(1):214-24. doi: 10.1046/j.1365-2958.2001.02376.x. PMID: 11298288.

Supplementary Table 2: Primers used in this study.

Primer name	Sequence 5' - 3'	Function
Kan F	ATTCTCCTTGGTTCTCATGTTTGACAGCTTAT	Amplification of the kanamycin resistance cassette from pJMK30 for knock-out mutagenesis cloning
Kan R	GCACACCTTGGCTAGGTACTAAAACAATTCAT	"
Cat F	ATTCTCCTTGGTTCTCATGTTTGACAGCTTGAATTCCTGCAGCCCGGGGG	Amplification of the chloramphenicol resistance cassette from pAV35 for knock-out mutagenesis cloning
Cat R	GCACACCTTGGCTAGGTACTAAAACAATTCACTAGTGGATCCCGGGTACC	"
Apr F	ATTCTCCTTGGTTCTCATGTTTGACAGCTTCGTAACAAGGTAACCGTAGG	Amplification of the apramycin resistance cassette from pRRA for knock-out mutagenesis cloning
Apr R	GCACACCTTGGCTAGGTACTAAAACAATTCTTACTTTGTACTCTAGGGCC	"
cj0903c F1	GAGCTCGGTACCCGGGGATCCTCTAGAGTCATAGGTAGAATGTGCTTTAATG	Amplification of upstream flank for knock-out mutagenesis cloning
cj0903c R1	AAGCTGTCAAACATGAGAACCAAGGAGAATAATATCAGACGCTTTATTGG	"
cj0903c F2	GAATTGTTTTAGTACCTAGCCAAGGTGTGCTTAATGCTAGCAAACTTGG	Amplification of downstream flank for knock-out mutagenesis cloning
cj0903c R2	AGAATACTCAAGCTTGCATGCCTGCAGGTCAAATTACTTCTTGCTAAGGTG	"
cj0903c pC46 F	AATATTCGTCTCACATGAATTTAGATATTATGCTAGATTTTG	Amplification for insertion into pC46 complementation vector
cj0903c pC46 R	AATATTCGTCTCACATGTTTGAAAATCCTAGCAATG	"
cj0935-4c F1	GAGCTCGGTACCCGGGGATCCTCTAGAGTCAAGGATTTGCGTAAAGTG	Amplification of upstream flank for knock-out mutagenesis cloning
cj0935-4c R1	AAGCTGTCAAACATGAGAACCAAGGAGAATTACCGCTAAAATGAAACC	"
cj0935-4c F2	GAATTGTTTTAGTACCTAGCCAAGGTGTGCATTCGTTTTATATCGCCTATAG	Amplification of downstream flank for knock-out mutagenesis cloning

cj0935-4c R2	AGAATACTCAAGCTTGCATGCCTGCAGGTCGGTAGATCCATTAAGGTAATAGC	"
cj0552-4 F1	GAGCTCGGTACCCGGGGATCCTCTAGAGTCAATTGCCAATTTATGGATAC	Amplification of upstream flank for knock-out mutagenesis cloning
cj0552-4 R1	AAGCTGTCAAACATGAGAACCAAGGAGAATAAACAATCAAAGGTATAGAAGC	"
cj0552-4 F2	GAATTGTTTTAGTACCTAGCCAAGGTGTGCATATAGAAGATGATTATCTTGGG	Amplification of downstream flank for knock-out mutagenesis cloning
cj0552-4 R2	AGAATACTCAAGCTTGCATGCCTGCAGGTCCATAATTTATAGCCATAGCTCC	"
cj0467-9 F1	GAGCTCGGTACCCGGGGATCCTCTAGAGTCATTATCCTGCTAATGCTGTT	Amplification of upstream flank for knock-out mutagenesis cloning
cj0467-9 R1	AAGCTGTCAAACATGAGAACCAAGGAGAATCAGGAGCTTGAGCTAATATAA	"
cj0467-9 F2	GAATTGTTTTAGTACCTAGCCAAGGTGTGCCGAAAGAGCAAAGAAATTT	Amplification of downstream flank for knock-out mutagenesis cloning
cj0467-9 R2	AGAATACTCAAGCTTGCATGCCTGCAGGTCGACAACAAAAACTCTTAAATGG	"
cj0901-2 F1	GAGCTCGGTACCCGGGGATCCTCTAGAGTCTAAGATTCATCATACCTTCAAA	Amplification of upstream flank for knock-out mutagenesis cloning
cj0901-2 R1	AAGCTGTCAAACATGAGAACCAAGGAGAATTTTTTAATATTTCTATCGGGAA	"
cj0901-2 F2	GAATTGTTTTAGTACCTAGCCAAGGTGTGCAGATTGAGAGAATTTTTAAACAA	Amplification of downstream flank for knock-out mutagenesis cloning
cj0901-2 R2	AGAATACTCAAGCTTGCATGCCTGCAGGTCCAGTTTTAATTGATGTTGTTGT	"
cj0940 F1	GAGCTCGGTACCCGGGGATCCTCTAGAGTCTTGGTTGCAAGTTTAAATATAG	Amplification of upstream flank for knock-out mutagenesis cloning
cj0940 R1	AAGCTGTCAAACATGAGAACCAAGGAGAATTCTATCAAAGTCAAAAATAATCAC	"

cj0940 F2	GAATTGTTTTAGTACCTAGCCAAGGTGTGCACATATCTTGTTTTGACTTTACC	Amplification of downstream flank for knock-out mutagenesis cloning
cj0940 R2	AGAATACTCAAGCTTGCATGCCTGCAGGTCAAGACTCGAACTTATGACATC	"
cj0817 F1	GAGCTCGGTACCCGGGGATCCTCTAGAGTCAAGATGGAAGCTATTCTAAAGA	Amplification of upstream flank for knock-out mutagenesis cloning
cj0817 R1	AAGCTGTCAAACATGAGAACCAAGGAGAATGCACTCAAATTTAAAGCAA	"
cj0817 F2	GAATTGTTTTAGTACCTAGCCAAGGTGTGCGACATTAAAGAAGTTTTTAAGCA	Amplification of downstream flank for knock-out mutagenesis cloning
cj0817 R2	AGAATACTCAAGCTTGCATGCCTGCAGGTCAAAAGAATTACTAAGGCTGTTC	"
CJJ81176_0067 F1	GAGCTCGGTACCCGGGGATCCTCTAGAGTCGATGAATTTAACGCTTTTCT	Amplification of upstream flank for knock-out mutagenesis cloning
CJJ81176_0067 R1	AAGCTGTCAAACATGAGAACCAAGGAGAATCTGCTATAGCTGCATCTATG	"
CJJ81176_0067 F2	GAATTGTTTTAGTACCTAGCCAAGGTGTGCCACAAAAGAACCTTATATGGG	Amplification of downstream flank for knock-out mutagenesis cloning
CJJ81176_0067 R2	AGAATACTCAAGCTTGCATGCCTGCAGGTCGCTTGGAGTATTTAGCTTGA	"
CJJ81176_0492 F1	GAGCTCGGTACCCGGGGATCCTCTAGAGTCCGCATTTATAAAAGTACAGC	Amplification of upstream flank for knock-out mutagenesis cloning
CJJ81176_0492 R1	AAGCTGTCAAACATGAGAACCAAGGAGAATGCAGGAGCTTGAACTAATAT	"
CJJ81176_0492 F2	GAATTGTTTTAGTACCTAGCCAAGGTGTGCCATTATTTTGCCTTTGTCTT	Amplification of downstream flank for knock-out mutagenesis cloning
CJJ81176_0492 R2	AGAATACTCAAGCTTGCATGCCTGCAGGTCGTTGTACTTTTAATCATGCG	"
CJJ81176_0494 F1	GAGCTCGGTACCCGGGGATCCTCTAGAGTCTGTTATGCCACTTTTGGTAT	Amplification of upstream flank for knock-out mutagenesis cloning

CJJ81176_0494 R1	AAGCTGTCAAACATGAGAACCAAGGAGAATATGCGAACCATAATACTTTT	"
CJJ81176_0494 F2	GAATTGTTTTAGTACCTAGCCAAGGTGTGCGCTAAAGCTGTAGCAGATAA	Amplification of downstream flank for knock-out mutagenesis cloning
CJJ81176_0494 R2	AGAATACTCAAGCTTGCATGCCTGCAGGTCCAATAACAGACAACAAAAACTC	"
CJJ81176_0912 F1	GAGCTCGGTACCCGGGGATCCTCTAGAGTCCTTCATATCGTTAAACCAACT	Amplification of upstream flank for knock-out mutagenesis cloning
CJJ81176_0912 R1	AAGCTGTCAAACATGAGAACCAAGGAGAATGTCTGTATTTGGGACAATCT	"
CJJ81176_0912 F2	GAATTGTTTTAGTACCTAGCCAAGGTGTGCGCTGATTTGACTATGGCTTT	Amplification of downstream flank for knock-out mutagenesis cloning
CJJ81176_0912 R2	AGAATACTCAAGCTTGCATGCCTGCAGGTCGCATGGTATTTCAGCATTT	"
CJJ81176_0964 F1	GAGCTCGGTACCCGGGGATCCTCTAGAGTCCAGCCTTAGAGCTTGAGAA	Amplification of upstream flank for knock-out mutagenesis cloning
CJJ81176_0964 R1	AAGCTGTCAAACATGAGAACCAAGGAGAATGACTTTGATAGAGGTTTTAAAGG	"
CJJ81176_0964 F2	GAATTGTTTTAGTACCTAGCCAAGGTGTGCAGTGCGCAAGAGATAAATTC	Amplification of downstream flank for knock-out mutagenesis cloning
CJJ81176_0964 R2	AGAATACTCAAGCTTGCATGCCTGCAGGTCCCCTAACAATAAAAATGGTGTC	"

Supplementary Table 3: RNAseq significant DEGs.

Locus	T5.log2 Ratio	T20.log2 Ratio	T45.log2 Ratio	adj.p.value.T5	adj.p.value.T20	adj.p.value.T45	adj.F.p.val
cj0007	-1.78	-1.93	-2.26	2.E-10	8.E-11	1.E-11	3.E-11
cj0008	-2.24	-2.06	-2.19	3.E-14	8.E-14	4.E-14	3.E-14
cj0009	-2.35	-2.38	-2.51	2.E-12	2.E-12	8.E-13	1.E-12
cj0086c	-1.98	-1.76	-1.54	3.E-08	2.E-07	9.E-07	8.E-08
cj0154c	1.50	0.80	1.14	4.E-05	1.E-02	7.E-04	2.E-04
cj0204	-1.55	-1.58	-1.81	6.E-07	1.E-06	3.E-07	4.E-07
cj0501	-1.52	-1.80	-2.12	1.E-07	4.E-08	8.E-09	1.E-08
cj0553	-1.63	-1.70	-1.79	1.E-11	8.E-12	5.E-12	7.E-12
cj0554	-2.08	-2.07	-1.99	3.E-13	3.E-13	4.E-13	2.E-13
cj0699c	-3.10	-3.30	-3.48	2.E-12	1.E-12	7.E-13	1.E-12
cj0903c	-1.77	-1.74	-1.96	4.E-13	5.E-13	1.E-13	2.E-13
cj0934c	-2.83	-3.03	-2.84	1.E-11	7.E-12	1.E-11	8.E-12
cj0935c	-3.33	-3.36	-3.63	3.E-11	3.E-11	1.E-11	2.E-11
cj0987c	-2.05	-2.21	-2.24	4.E-10	2.E-10	2.E-10	2.E-10
cjp06	-0.57	-0.47	-1.89	4.E-01	5.E-01	1.E-02	4.E-02
cj1537c	-1.39	-1.06	-1.56	9.E-04	1.E-02	6.E-04	1.E-03
cj0241c	1.33	1.03	1.51	3.E-06	7.E-05	2.E-06	3.E-06
cj1664	0.68	0.96	1.65	4.E-03	5.E-04	3.E-06	1.E-05
cj1661	0.83	1.20	1.66	4.E-05	2.E-06	5.E-08	2.E-07
cj0753c	1.10	1.35	1.79	7.E-05	2.E-05	1.E-06	3.E-06

Supplementary Table 4: Distribution of *gutA* and *amt* in *Campylobacter* spp.

		***gutA* (*cj0903c*)**			***amt***		
**Species**	**Count**	**No Gene**	**Gene**	**Pseudo**	**No Gene**	**Gene**	**Pseudo**
C. anatolicus	3	3	0	0	3	0	0
C. armoricus	11	0	11	0	11	0	0
C. aviculae	1	0	1	0	1	0	0
C. avium	2	2	0	0	2	0	0
C. bilis	8	0	8	0	8	0	0
C. blaseri	3	3	0	0	3	0	0
C. californiensis	9	9	0	0	9	0	0
C. canadensis	8	0	8	0	8	0	0
C. coli	1685	24	1650	11	7	1671	7
C. concisus	232	1	231	0	232	0	0
C. corcagiensis	3	3	0	0	3	0	0
C. cuniculorum	3	0	2	1	0	3	0
C. curvus	16	16	0	0	16	0	0
C. devanensis	35	35	0	0	0	35	0
C. estrildidarum	1	0	1	0	1	0	0
C. felis	6	0	6	0	6	0	0
C. fetus	102	1	73	28	102	0	0
C. gastrosuis	1	1	0	0	0	1	0
C. geochelonis	4	4	0	0	4	0	0
C. gracilis	4	4	0	0	4	0	0
C. helveticus	29	0	29	0	29	0	0
C. hepaticus	44	0	44	0	44	0	0
C. hominis	4	4	0	0	4	0	0
C. hyointestinalis	59	59	0	0	59	0	0
C. iguaniorum	3	3	0	0	3	0	0
C. infans	1	1	0	0	0	1	0
C. insulaenigrae	19	0	19	0	19	0	0
C. jejuni	3257	14	3234	9	1174	1869	214
C. lanienae	30	30	0	0	0	30	0
C. lari	160	0	160	0	160	0	0
C. magnus	5	0	5	0	5	0	0
C. majalis	1	1	0	0	1	0	0
C. massiliensis	1	0	1	0	1	0	0
C. mucosalis	6	6	0	0	6	0	0
C. novaezeelandiae	6	0	6	0	6	0	0
C. ornithocola	2	0	2	0	2	0	0
C. peloridis	5	0	5	0	5	0	0
C. pinnipediorum	11	11	0	0	11	0	0
C. porcelli	6	6	0	0	0	6	0
C. portucalensis	1	1	0	0	1	0	0
C. rectus	4	0	4	0	4	0	0
C. showae	12	0	12	0	12	0	0

C. sp.	278	61	217	0	200	66	12
C. sputorum	17	17	0	0	17	0	0
C. subantarcticus	3	0	3	0	3	0	0
C. suis	1	0	1	0	1	0	0
C. taeniopygiae	1	0	1	0	1	0	0
C. troglodytis	1	0	1	0	0	1	0
C. upsaliensis	152	0	151	1	152	0	0
C. ureolyticus	33	33	0	0	33	0	0
C. vicugnae	12	12	0	0	5	7	0
C. volucris	16	0	16	0	16	0	0
C. vulpis	16	0	16	0	16	0	0
